# 861. Patterns of Injury for Burn Patients Involved in Civilian Explosion or Combustion Mechanisms

**DOI:** 10.1093/jbcr/irag033.331

**Published:** 2026-04-06

**Authors:** Ashleigh Bull, Natalie Nguyen, Colette Galet, Alexander Kurjatko

**Affiliations:** University of Iowa Hospitals and Clinics, Iowa City, IA; Baylor University Medical Center, Dallas, TX; University of Iowa, Iowa City, IA; University of Iowa, Iowa City, IA

## Abstract

**Introduction:**

Burn injuries can occur from the combustion or explosion of volatile materials. Explosions in the military setting are known to often be complicated by the presence of polytrauma, but may not be as prevalent in the civilian setting. We aimed to characterize the pattern of non-burn traumatic injuries (NBTI) sustained by patients injured by such mechanisms in the civilian setting.

**Methods:**

This is a single-center retrospective cohort study of adult patients with explosion and combustion-related burn injuries admitted between July 2015 and March 2024. Medical record review identified seven categories of explosive or combustive injury mechanisms. Data collected included demographics, comorbidities, burn injury information, Injury Severity Score (ISS), clinical factors, and outcomes. Categorical variables were compared using Chi-square or Fisher exact tests, and continuous variables using Mann–Whitney U tests. *p* value <0.05 was considered statistically significant.

**Results:**

The cohort included 493 patients. Patients were predominantly male (80.3%) and Caucasian (92.9%). The mean total body surface area burned was 7%. Inhalation injury was present in 8.9% of patients. NBTI were identified in 63 patients (12.8%). The most common NBTI consisted of superficial ocular injuries (58.7%), followed by lacerations/abrasions (34.9%), and orthopedic injuries (20.6%). About 32% of NBTI required a procedure, with most such incidences stemming from firework blasts. NBTI had higher ISS (5 [2-14.5] vs. 1 [1-4], *p*<.001), were more likely to be on a ventilator (38.1% vs. 23.8%, *p*=.02), hypothermic (36.6 [36-37.1] vs. 36.8 [36.4-37.1], *p*=.034), and had lower Glasgow Coma Scale (GCS) Scores (15 [7-15] vs. 15 [15-15], *p*=.002) on arrival. Mechanisms that fell under the category of “explosion of flammable liquids” were less likely to result in NBTI (47.2% vs. 20.6%), while mechanisms that fell under the category of “explosion with chemical combustion” (4.4% vs. 28.6%) and “indoor mechanical explosions” (5.3% vs 22.2%) were more likely to result in NBTI (*p*<.001). In our setting, burn patients may bypass the emergency department (ER) to the burn unit at the burn physician’s discretion. Patients who had NBTI were evaluated in the ER prior to arrival to the burn unit (39.7% vs. 5.1%, *p*<.001), and had trauma evaluation (34.9% vs. 2.8%, *p*<.001) more often than their counterparts.

**Conclusions:**

Burn mechanisms involving explosion or combustion may result in NBTI. However, injuries that require intervention are relatively uncommon. Patients with NBTI were more likely to have higher ISS, require ventilation, be more hypothermic, have lower GCS scores, and fall under specific mechanism categories.

**Applicability of Research to Practice:**

ER and trauma triage rates may be improved by utilizing risk factors to predict the presence of NBTI.

**Funding for the study:**

N/A.

